# Co-occurrence of posterior chest wall pilonidal sinus with melanocytic nevus: a challenging presentation: a case report

**DOI:** 10.1186/s13019-024-02802-y

**Published:** 2024-06-12

**Authors:** Abdulwahid M. Salih, Fahmi Hussein kakamad, Aso S. Muhialdeen, Hardi M. Zahir, Yadgar A. Saeed, Halkawt O. Ali, Sara N. Ahmad, Marwan N. Hassan, Shko H. Hassan, Mohammed Subhan Mohammed

**Affiliations:** 1 Smart Health Tower, Madam Mitterrand Street, Sulaimani, Kurdistan Iraq; 2 College of Medicine, University of Sulaimani, HC8V+F66, Madam Mitterrand Street, Sulaimani, Kurdistan 46001 Iraq; 3 Kscien Organization, Hamdi Street, Azadi Mall, Sulaimani, Kurdistan Iraq; 4 Research Center, University of Halabja, Halabja, Kurdistan Iraq

**Keywords:** Nevus, Chest, Pilonidal sinus, Hair

## Abstract

**Introduction:**

To date, only a limited number of case reports have documented the co-occurrence of PNS and melanocytic nevus in the medical literature. This study aims to report an exceptionally rare case of posterior chest wall PNS in conjunction with a melanocytic nevus.

**Case presentation:**

A 46-year-old female presented with a long-standing black lesion on her left upper posterior chest wall, that had become painful in the two months prior to presentation. There was a painful, dark blue, non-erythematous, and non-tender nodule on the left upper posterior chest wall. Based on the patient’s desire for cosmetic purposes, the lesion was excised totally with primary closure under local anaesthesia. Histopathological examination revealed intradermal melanocytic nevus with inflamed pilonidal sinus.

**Discussion:**

The rarity of posterior chest wall PNS associated with nevi poses unique diagnostic and therapeutic challenges for clinicians. The distinct anatomical location, different from the conventional region, and the rare association between the two conditions may delay accurate diagnosis and result in mismanagement or inappropriate interventions.

**Conclusion:**

The posterior chest wall PNS is another type of atypical PNS that is extremely rare. The association between PNS and blue nevus is a fascinating medical finding that deserves further investigation.

## Introduction

Pilonidal sinus disease (PNS) is a chronic inflammatory disorder associated with hair ingrowth into the skin [1–3]. Although PNS predominantly affects the sacrococcygeal area, it can also manifest in various atypical sites such as the umbilicus, intermammary, suprapubic, groin, axilla, face, hand, postauricular, preauricular, submental, clitoris, scalp, and endoanal [4–7]. Overall, the condition is more common in the developing countries [8]. The occurrence of a PNS on the chest wall is an unusual condition that has received little attention in the medical literature. Although anterior chest wall PNS has been sporadically reported, the occurrence of a PNS on the posterior chest wall remains an uncommonly documented condition [9]. Similarly, melanocytic nevus, characterized by benign pigmented lesions, is prevalent in specific body regions such as the buttocks and sacrococcygeal area [10]. To date, only a limited number of case reports have documented the co-occurrence of PNS and melanocytic nevus in the medical literature [11].

This study aims to present an exceptionally rare case of posterior chest wall PNS in conjunction with a melanocytic nevus.

## Case presentation

### Patient’s information

A 46-year-old female presented with a long-standing black lesion on her left upper posterior chest wall that had become painful in the two months prior to the presentation to the clinic. She was multiparous and non-smoker. No remarkable Past medical and surgical history. Negative history of PNS at the other sites of her body. The patient did not complain of pain, drainage, or other symptoms.

### Clinical findings

There was a painful, dark blue, non-erythematous, and non-tender nodule on the left upper posterior chest wall. The rest of the physical examination was unremarkable.

### Diagnostic assessment

The patient was diagnosed clinically as having a nevus, and no investigation was requested.

### Therapeutic intervention

**Fig. 1:**
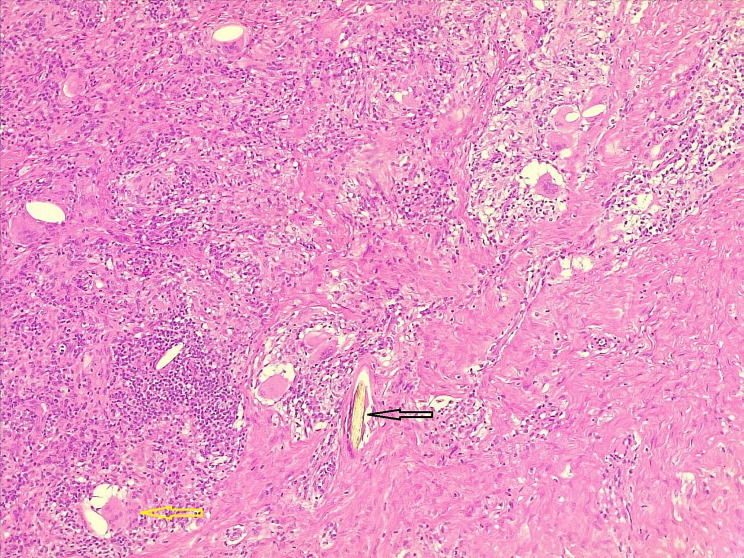
Hair shaft (black arrow), surrounded by mixed inflammatory cells with multinucleated giant cells (yellow arrow)

**Fig. 2:**
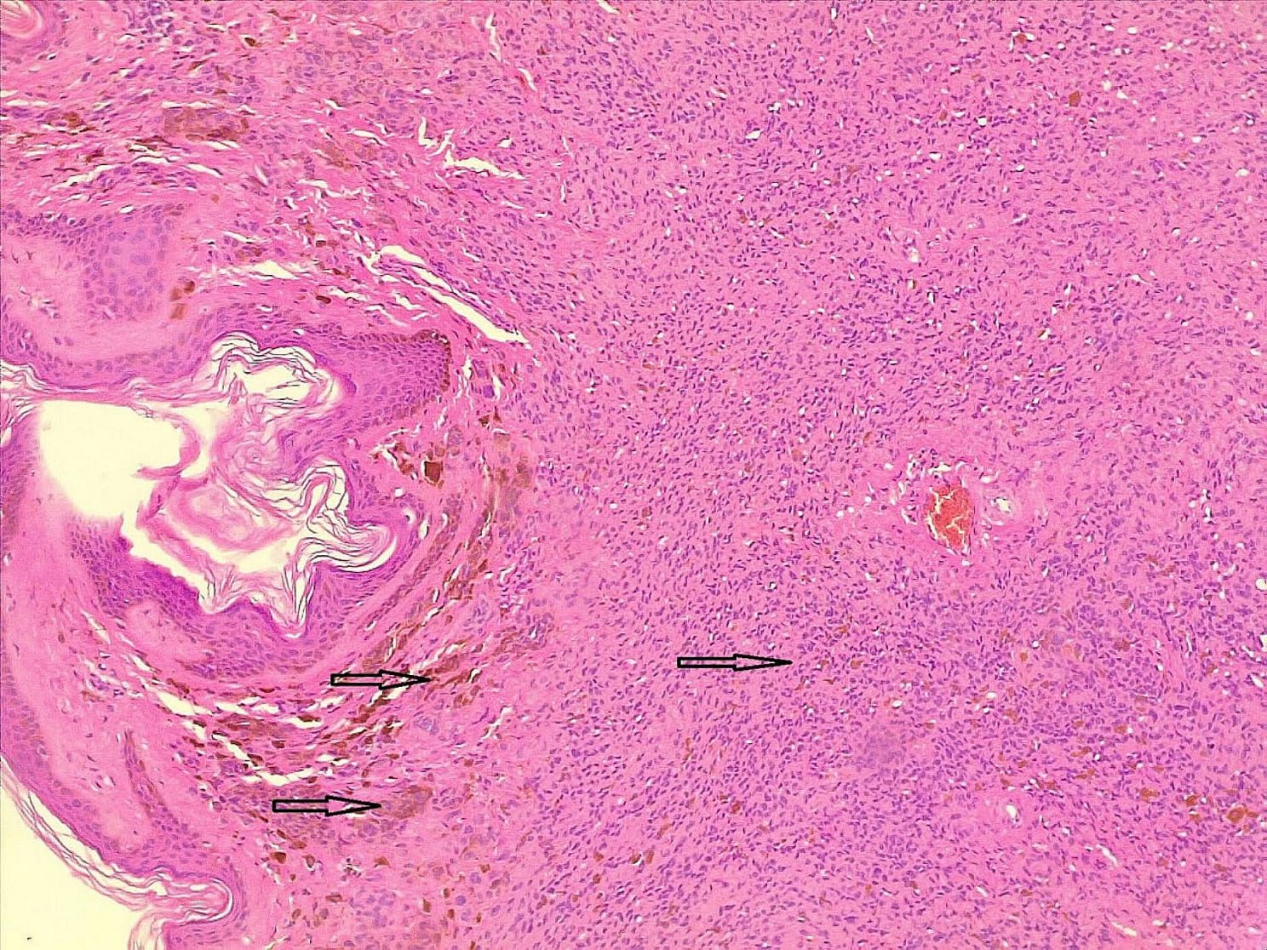
Dermis occupied by benign-looking melanocytic cells with melanin pigment (dark arrows)

Based on the patient’s desire for cosmetic purposes, the lesion was excised totally with primary closure under local anesthesia. Histopathological examination revealed an intradermal melanocytic nevus with an inflamed PNS (Figs. 1 and 2).

### Follow-up

Postoperative follow up period was uneventful. There were no recurrences of the PNS throughout the three-month follow-up period.

## Discussion

PNS often appears as an abscess with or without sinuses in the middle of the natal cleft. It is a surgical-dermatological condition that is causing an increase in the frequency of surgical procedures, particularly among young adults [11, 12]. Although it is a common condition that can affect people of all ages, it is unusual in the extremities [13]. Males are more commonly affected than females, which may be associated with sex hormones and their more hirsute nature [1, 14]. The incidence of PNS has grown over the last 50 years, with the frequency in Germany increasing from 29/100,000 in 2000 to 48/100,000 in 2012 [12, 15]. In Asian countries, the occurrence of PNS is notably scarce, leading to a lack of epidemiological data on the disease. Despite this, a study noted an incidence rate of approximately 0.07% for PNS in 2017 [7]. Overall, the increasing incidence of pilonidal sinus over time may be multifactorial, influenced by a combination of improved diagnosis, changing lifestyles, environmental factors, genetic predisposition, population growth, and enhanced awareness.

Although the PNS can occur in any other part of the body, its appearance on the posterior chest wall is an infrequently described presentation of this well-known condition [5, 7, 9]. Melanocytic nevi are benign lesions caused by the clonal proliferation of a melanocyte. It is expected that this change would occur either spontaneously or in response to extrinsic stimuli (for example, sun exposure), resulting in histologically symmetric, essentially uniform cell proliferation with homogenous alterations affecting the majority of component cells [16]. A hairy nevus can become irritated and give rise to an inflammatory reaction. This reaction may occur due to various factors such as friction, trauma, or exposure to certain irritants. Additionally, hair follicles within the nevus can become ingrown, leading to inflammation and irritation. However, it’s essential to note that not all hairy nevi will necessarily cause irritation or inflammation, and the severity of the reaction can vary depending on individual factors and the specific characteristics of the nevus [17].

The etiology of PNS is unknown; however, it is believed to be caused by an accumulation of hair and skin debris in the natal cleft, which can lead to infection and the creation of a cyst or sinus tract [1, 3, 16]. Some potential risk factors have been identified, including male gender, positive family history, particularly first-degree relatives, obesity, prolonged sitting periods exceeding 6 h daily, poor personal hygiene (bathing frequencies of 2 or fewer times per week), an inactive lifestyle, excess body hair, and a deep natal cleft [1, 5, 7, 18, 19]. Recurrence rates of 7–42% have been reported after primary closure [1, 19].

.

Clinical presentation is the primary method for diagnosing PNS. Ultrasonography can also detect hair strands within the lesion, while fine-needle aspiration biopsy offers another diagnostic option [20]. PNS may be asymptomatic or present with pain, redness, swelling, tenderness, abscess formation, or drainage of pus or blood [1, 3]. Nevus typically manifests as a nodular growth in young adulthood with limited progression, though some may develop into melanoma [20]. Nevus diagnosis can involve various methods, including clinical examination, dermoscopy, and sometimes biopsy. Clinical examination involves visual inspection of the skin lesion, noting its color, size, shape, and other characteristics. Dermoscopy, a non-invasive technique, allows for a closer examination of the lesion’s surface features and structures. In cases where the diagnosis is uncertain or further information is needed, a histological examination considered as a gold standard diagnosis [21]. The clinical diagnosis of the current case can be challenging, especially when encountering unusual presentations, pain, or tenderness, emphasizing the need for vigilance among healthcare professionals.

Eradication of the sinus tract, complete healing, and prevention of recurrence are the main three principles in the management of PNS [1]. The ideal treatment should be effective, safe, low cost, and minimize discomfort, hospital stay, and recurrence rate [1, 22]. Surgical excision of the PNS is the primary treatment, but special care must be taken during excision to avoid compromising the blue nevus. Regular follow-up is crucial to monitor both conditions and detect any potential complications or recurrences. The risk of wound infection following surgery is significant, particularly from anaerobic bacteria, which causes delayed wound healing [13, 23]. The current case was diagnosed clinically as a blue nevus and managed with surgical excision under local anesthesia.

The association between PNS and blue nevus is not fully understood, but it may involve congenital skin weaknesses or changes in the local environment, possibly influenced by shared genetic factors or embryological origins [10]. This rarity presents unique challenges for diagnosis and treatment, often leading to delays and potential mismanagement. Comprehensive documentation and analysis of such cases are needed to improve medical understanding and patient care. This study contributes valuable insights into this rare association, aiding clinicians in diagnosis and management.

In conclusion, the posterior chest wall PNS is another type of atypical PNS that is extremely rare. The association between PNS and blue nevus is a fascinating medical finding that deserves further investigation. Although rare, it underscores the complexity of dermatological and surgical interactions. Continued research into the underlying mechanisms of this association can help improve our understanding of both conditions and guide optimal management strategies for affected patients.

## Data Availability

The datasets used and/or analyzed during the current study are available from the corresponding author on reasonable request.
